# Factors Motivating Individuals to Consider Genetic Testing for Type 2 Diabetes Risk Prediction

**DOI:** 10.1371/journal.pone.0147071

**Published:** 2016-01-20

**Authors:** Jennifer Wessel, Jyoti Gupta, Mary de Groot

**Affiliations:** 1 Department of Epidemiology, Indiana University Richard M. Fairbanks School of Public Health, Indianapolis, IN, United States of America; 2 Department of Medicine, Indiana University School of Medicine, Indianapolis, IN, United States of America; McMaster University, CANADA

## Abstract

The purpose of this study was to identify attitudes and perceptions of willingness to participate in genetic testing for type 2 diabetes (T2D) risk prediction in the general population. Adults (n = 598) were surveyed on attitudes about utilizing genetic testing to predict future risk of T2D. Participants were recruited from public libraries (53%), online registry (37%) and a safety net hospital emergency department (10%). Respondents were 37±11 years old, primarily White (54%), female (69%), college educated (46%), with an annual income ≥$25,000 (56%). Half of participants were interested in genetic testing for T2D (52%) and 81% agreed/strongly agreed genetic testing should be available to the public. Only 57% of individuals knew T2D is preventable. A multivariate model to predict interest in genetic testing was adjusted for age, gender, recruitment location and BMI; significant predictors were motivation (high perceived personal risk of T2D [OR = 4.38 (1.76, 10.9)]; family history [OR = 2.56 (1.46, 4.48)]; desire to know risk prior to disease onset [OR = 3.25 (1.94, 5.42)]; and knowing T2D is preventable [OR = 2.11 (1.24, 3.60)], intention (if the cost is free [OR = 10.2 (4.27, 24.6)]; and learning T2D is preventable [OR = 5.18 (1.95, 13.7)]) and trust of genetic testing results [OR = 0.03 (0.003, 0.30)]. Individuals are interested in genetic testing for T2D risk which offers unique information that is personalized. Financial accessibility, validity of the test and availability of diabetes prevention programs were identified as predictors of interest in T2D testing.

## Introduction

In the last decade over 140 novel genetic markers robustly associated with type 2 diabetes (T2D)[1] and related traits[2] have been discovered. These discoveries are the building blocks of personalized medicine–using patient genetic risk information to guide prevention, diagnosis or treatment. A scientific framework has been proposed to evaluate genetic testing; the ACCE model (analytic validity, clinical validity, clinical utility and the ethical, legal or social implication)[3]. The clinical validity of genetic markers adds only marginal value in predicting future development of T2D [4, 5], and have been shown to be independent of family history[6, 7]. The emerging scientific evidence for the clinical utility of genetic testing (genetic test results are shown to improve health outcomes) for common diseases has shown mixed results[8] and only one has been conducted for T2D[9].

Few studies have examined the characteristics and reasons associated with interest in genetic testing for T2D risk[10]. Studies representing a broad range of common diseases found that the characteristics associated with reluctance to pursue genetic testing included: anxiety about genetic testing; older age; lower levels of education; and high perceived discrimination from employers and insurers[11–14]. Evenmore studies have indicated high genetic risk for a given disorder is associated with improved motivation to adopt a healthier lifestyle[9–11, 15–18]. Specific to T2D, participants reported greater knowledge of genetics, high perceived risk of T2D, and high motivation to adopt a healthier lifestyle were positively correlated with willingness to obtain T2D genetic testing[9]. Nonetheless participants in these studies have been limited to either high-risk individuals, members of managed care organizations, or have been predominantly well educated or white, non-Hispanic which has limited the generalizability of these findings to populations representing the full spectrum of risk for T2D (i.e. no risk to high risk). Moreover, studies have been limited in the breadth of psychosocial constructs measured in their study population; primarily measuring factual knowledge of diseases or genetics, or knowledge and attitudes of the benefit, consequence or intention to undergo genetic testing.

Through direct-to-consumer venues, genetic susceptibility testing for polygenic disorders such as T2D have become available to the public. Genetic susceptibility testing offers personalized and detailed risk information, which can also provide an opportunity to inform the public about T2D development and prevention. The current research examined extensive attitudinal factors affecting individuals’ decision to engage in genetic testing for T2D risk prediction in a socioeconomically diverse population. The purpose of the current study was to identify predictors of interest in genetic testing. We pose this question as a first step in understanding ways this new risk factor identification tool maybe relevant to patients.

## Materials and Methods

### Participants

This study was approved by Indiana University Institutional Review Board (1205008781). All participants provided written consent to participate in this study. Participants (n = 649) were residents of Indiana between the ages of 18–64 years, and were recruited June-July 2012 from 14 public libraries across the greater Indianapolis metropolitan area (n = 344; 53% of total sample), the emergency department (ED) of Wishard Hospital (n = 62; 10%), and online (n = 243; 37%) through a volunteer research participant registry (INResearch.org). To recruit participants from public libraries or the ED, research assistants set up tables located inside the library or the waiting room of the ED. Visitors of the public libraries or the ED were asked if they would be interested in filling out a survey on genetic testing. Research assistants were at the sites during peak hours for 1–2 days per library and 2 days at the ED. Participants of the volunteer registry received an email describing the study with a link to the online survey. To oversample groups that are often underrepresented in genetic testing (e.g. African-Americans, lower socioeconomic status) the ED of Wishard Hospital was chosen. It is the safety net health care system serving greater Indianapolis.

### Questionnaire

The questionnaire was designed using standard survey development procedures[19]. An extensive literature search was conducted on the assessment of attitudes, beliefs and behaviors associated with genetic testing in T2D and other chronic diseases to guide the questionnaire design. An initial sample of 75 items were created and distributed to five experts in the fields of psychology, sociology and anthropology for evaluation. Following revisions, items were piloted in a focus group of N = 10 participants to assess clarity of wording. Based on feedback, items were then edited resulting in a total of N = 59 items in the domains of demographic, personal and family medical history, psychosocial and behavioral factors related to interest in genetic testing for T2D risk prediction (S1 Appendix).

#### Demographic Characteristics

Age, gender, marital status, household income, education, race, ethnicity, and type of health insurance data were gathered. Questions were adapted from the 2010 U.S Census Bureau.

#### Personal and family health history

Participants answered questions on their personal medical history by checking boxes listing 9 conditions related to diabetes, hypertension, high cholesterol, heart disease, overweight/obese. Family history of T2D was measured with ‘Does type 2 diabetes run in your family (blood relatives)?’ using a yes/no/don’t know response format.

#### Psychosocial

Participants answered questions drawn from behavior change models [20] which included perceived health, knowledge of T2D, perceived risk and worry of developing T2D, awareness of genetic testing, trust of genetic results, motivation for and utility of genetic testing, barriers to genetic testing, attitudes towards genetic testing and T2D prevention, intention for genetic testing, worry related to genetic testing procedure and results, motivation to lower risk of T2D through lifestyle changes if genetic test results indicated high risk, and knowledge of basic genetics. Many of the questions were adapted from state-level Behavioral Risk Factor Surveillance Systems (BRFSS) questionnaires. Questions relating to worry were measured using a seven-point Likert scale (“Not worried at all” to “Extremely worried”). Other items had a five-point Likert scale (e.g. “Strongly agree”, “Agree”, “Neither agree nor disagree”, “Disagree”, “Strongly disagree”) or multiple choice response format.

#### Perceived health

We measured perceived health with the following item: ‘In general, you would describe your health as’ rated as Excellent/Very Good/Good/Fair/Poor[21].

#### T2D knowledge

We measured knowledge of T2D with three questions. The first two questions asked participants to check all boxes that applied. The first question asked ‘Which options do you feel are true for type 2 diabetes?: type 2 diabetes can be cured, type 2 diabetes can be prevented, type 2 diabetes is entirely inherited, type 2 diabetes is partly inherited and partly due to lifestyle conditions, the risk of getting type 2 diabetes, cannot be changed, the risk of getting type 2 diabetes can be changed, onset of type 2 diabetes can be delayed’. The second question was ‘Which options do you think can delay the onset of type 2 diabetes?: healthier eating, regular exercise, medication, cannot delay the onset of type 2 diabetes, other’. The third question was ‘If you get type 2 diabetes, then would you have an increased risk for other diseases too?’ Yes/not sure/no.

#### Perceived risk and worry of T2D

We measured perceived risk and worry of developing T2D with one question for each, respectively, using the same general format: ‘On a scale of 1 to 7, how [likely do you think / worried are you that] you will get type 2 diabetes in the future?’.

#### Worry

We also measured worry with six questions using the same response format: ‘On a scale of 1 to 7, how worried are you ….?’. The questions related to having genetic testing for T2D, cost of testing, collecting a sample with saliva or with blood, results predict a high risk or predict a low risk.

#### Awareness of genetic testing

We measured awareness of genetic testing with two questions on whether participants had ever heard of genetic testing (yes/no) and where they had heard of genetic testing by checking all boxes listing media, physician, family, friends, teachers, other.

#### Trust

We measured trust in genetic testing by asking ‘If a genetic test shows that you are at high risk for getting type 2 diabetes, you believe your actual risk for type 2 diabetes would be: high, medium, low, none’.

#### Motivation

Motivation to have genetic testing for T2D risk assessment was measured with three questions. The first question simply asked ‘your motivation to have genetic testing for type 2 diabetes is: very high/high/average/low/very low’. For the next two questions, participants could check all boxes that applied. The questions were reasons for currently being motivated (know someone with diabetes complications, family history, had diabetes during pregnancy, diabetes is preventable, future treatment, know risk before development, currently high risk, other) and options that would increase motivation (insurance covers cost, testing is not expensive, pre-/post-test counseling is available, test is accurate, physician recommends, family member with high genetic test results, other).

#### Attitudes

We measured attitudes towards genetic testing with two questions. We asked participants to identify reasons for rejecting genetic testing for type 2 diabetes, which included low risk, no family history, genetic risk is not changeable, prefer cancer testing, testing would be personally harmful, would be more worried if results showed high risk, no access to facilities and services, do not trust results, no time, other. We asked ‘Assuming that type 2 diabetes is completely preventable, would you now want genetic testing done for type 2 diabetes?’ using a yes/no/not sure response format.

#### Utility

We measured the perceived utility of genetic testing results by asking ‘Knowing your type 2 diabetes genetic test results will be useful to you.’ Participants could check all boxes that applied from a list of 7 items: uncertainty of risk would be clearer, can discuss results with physician, can convince your family to be tested, can better prepare yourself for the future, test results will motivate you to take actions to decrease risk, wanting to know results so their children could also be tested, other.

#### Barriers to Genetic Testing

We measured barriers to genetic testing by asking participants ‘Assuming genetic testing is free of cost, would you now want genetic testing done for type 2 diabetes?’ Yes/no/not sure.

#### Genetic literacy

Participants answered five true/false questions that assessed general knowledge of genetics. Questions were modified from existing measures of genetic literacy[16, 22, 23]. One point was given to each question answered correctly and summed to create a genetics knowledge total score (range 0–5). A score of four or greater indicated high levels of genetic knowledge.

### Procedures

Questionnaires were self-administered using paper or web-based versions available in English or Spanish (1.8% of total surveys). The online questionnaire was distributed to prospective participants registered at Indiana’s volunteer research participant registry (INResearch.org). The printed version of the questionnaire was handed to eligible participants by trained interviewers at the public libraries or the ED waiting room of Wishard hospital in Indianapolis. After completing the survey, each study participant was compensated for his or her time with a $10 gift card. After completing the survey, participants were offered educational materials describing diabetes, risk factors and symptoms, and the Genetic Information Non-discrimination Act (GINA bill). All materials were available in both English and Spanish.

### Data Analysis

For all statistical analyses, SAS software version 9.4, was used. Individuals who self-reported diabetes (n = 50) and BMI<15 kg/m^2^ (n = 1) were excluded from the sample. BMI was categorized using standard definitions: underweight/normal (BMI<25.0 kg/m^2^), overweight (25.0–29.9 kg/m^2^), or obese (>30 kg/m^2^). The sample was grouped by interest in pursuing T2D genetic testing (“Would you like to get genetic testing done for type 2 diabetes?” yes vs. no or not sure). Differences in demographic and psychosocial variables by group were evaluated using univariate logistic regression analyses. Bonferroni correction was applied for the number of tests performed (Bonferroni correction = 0.05/70 = 0.0007). Multivariable logistic regression analyses were computed to predict willingness to engage in T2D genetic testing (yes) adjusted for age, gender, recruitment location and BMI (as a continuous variable). Variables that showed a significant relationship in the univariate models (p < .0007) were entered into the multivariable models if they were not multicollinear (i.e. Pearson and Spearman Correlations ≤ .40) or conceptually the same as the outcome variable.

## Results

### Participant Characteristics

Participants (n = 598) had a mean age of 37±11 years, were primarily White (54%), female (69%), had no college degree (54%), private health insurance (52%) and an annual household income of $25,000 or greater (56%; Table 1). The majority of participants self-reported BMI values as being overweight (27%) or obese (35%) and a family history of T2D (42%). Eighty-one percent of respondents agreed or strongly agreed with the statement ‘genetic testing should be available to the public.’ Fifty-seven percent of participants reported knowledge that T2D is preventable.

**Table 1:** Demographic Characteristics of the Study Population (n = 598).

	Interest in T2D Genetic Testing^a^			
Characteristic	Total	Yes (n = 312)	No (n = 285)	p-value
Age (years)	37±11	37±11	37±12	0.5918
Females	410 (68.9)	226 (72.4)	184 (65.0)	0.0509
BMI (kg/m2)	29.0±7.8	29.9±8.3	28.2±7.1	0.0085
Family History of T2D (Yes)	244 (41.6)	150 (49.2)	94 (33.5)	0.0001*
**Race**				
White	307 (54.1)	161 (55.1)	146 (52.9)	0.5443
Black	217 (38.2)	106 (36.3)	111 (40.2)	
Asian/Latino/Native American	44 (7.8)	25 (8.6)	19 (6.9)	
**Marital Status**				
Single	337 (56.5)	168 (54.0)	168 (59.2)	0.0054
Married	201 (33.8)	101 (32.5)	100 (35.2)	
Cohabitating	58 (9.8)	42 (13.5)	16 (5.6)	
**Education**				
College or more	269 (45.9)	128 (42.2)	141 (49.8)	0.1948
Some college	146 (24.9)	83 (27.4)	63 (22.3)	
Completed high school	98 (16.7)	56 (18.5)	42 (14.8)	
Some/less high school	73 (12.5)	36 (11.9)	37 (13.1)	
**Annual Household Income**				
($) <25,000	255 (43.6)	134 (44.4)	121 (42.8)	0.6891
25,000–50,000	163 (27.9)	88 (29.1)	75 (26.5)	
50,001–75,000	59 (10.1)	26 (8.6)	33 (11.7)	
75,001–100,000	47 (8.0)	25 (8.3)	22 (7.8)	
>100,000	61 (10.4)	29 (9.6)	32 (11.3)	
**Health Insurance**				
Commercial	300 (51.8)	159 (53.2)	141 (50.4)	0.1137
Medicare	38 (6.6)	16 (5.4)	22 (7.9)	
Medicaid	86 (14.9)	52 (17.4)	56 (45.2)	
None	155 (26.8)	72 (24.1)	83 (29.6)	
**Recruitment Location**				
Public Library	316 (53.0)	146 (46.8)	170 (59.9)	0.0025
Web survey	225 (37.8)	138 (44.2)	87 (30.6)	
Emergency Department	55 (9.2)	28 (9.0)	27 (9.5)	

Results are presented as mean±standard deviation or frequency.

^*a*^Comparison between yes versus no interest in genetic testing.

*P-value remains significant after Bonferonni correction; p<0.007.

### Factors Influencing Decisions to Engage in T2D Genetic Testing: Univariate Analyses

Approximately half (52%) of the respondents indicated willingness to participate in genetic testing to predict their T2D risk. Interest in genetic testing did not differ by age, gender, BMI, race, marital status, education, annual household income levels, and health insurance (p’s>.0007; Table 1). Individuals with a family history of T2D (48.1%) were more likely to report an interest in T2D genetic testing (p = 0.0001; Table 1). The majority of respondents scored high on questions pertaining to basic knowledge of genetics (86.8%), and were conceptually aware of genetic testing (62.5%) despite having no prior personal experience with genetic testing (89.9%; Table 2). Genetic literacy, however, was not significantly associated with willingness to engage in T2D genetic testing (p = 0.3; Table 2). Most adults believed they were in excellent/very good/good health (82.5%), with a low (47%) or neutral risk (28%) of developing T2D in the future (Table 2). Individuals who perceived themselves as having low risk of developing T2D were significantly less interested in genetic testing (p = 0.0004). Worry about perceived risk of developing T2D significantly predicted the decision to consider genetic testing for T2D risk (p = 0.0002).

**Table 2:** Univariate Analyses of Psychosocial Factors Predicting Willingness to Engage in Genetic Testing for Type 2 Diabetes Risk Prediction (n = 598).

	Interest in T2D Genetic Testing			
Psychosocial Factor	Total	Yes (n = 312)	No (n = 285)	p-value
**General Health: Good/Very/Excellent**	495 (82.5)	245 (78.2)	249 (87.2)	0.0038
**Risk Perception**				
Perceived T2D Risk: High	136 (23.3)	86 (28.3)	50 (17.9)	0.0004*
Neither	166 (28.4)	94 (30.9)	72 (25.7)	
Low	282 (48.3)	124 (40.8)	158 (56.4)	
**Awareness**				
Genetic Testing is Available	370 (62.5)	195 (62.9)	175 (62.1)	0.8317
Source: Media	364 (61.1)	193 (61.9)	119 (38.1)	0.6803
Source: Physician	195 (32.7)	116 (37.2)	79 (27.8)	0.0150
Source: Family	185 (31.1)	99 (31.8)	86 (30.3)	0.6831
Source: Friends	108 (18.1)	57 (18.3)	51 (18.0)	0.9215
Source: Teachers	106 (17.8)	54 (17.3)	52 (18.3)	0.7493
**Knowledge**				
T2D is Curable	104 (17.5)	48 (15.4)	56 (19.7)	0.1638
T2D is Preventable	342 (57.4)	188 (60.3)	154 (54.2)	0.1370
T2D is Inherited	53 (8.9)	28 (9.0)	25 (8.8)	0.9414
T2D is both Inherited and due to Lifestyle Conditions	376 (63.1)	210 (67.3)	166 (58.5)	0.0252
T2D Risk Can not be Changed	28 (4.7)	13 (4.2)	15 (5.3)	0.5205
T2D Risk Can be Changed	315 (52.9)	175 (56.1)	140 (49.3)	0.0970
Onset of T2D Can be Delayed	262 (44.0)	134 (43.0)	128 (45.1)	0.6022
T2D Onset Can be Delayed with Healthier Eating	532 (89.3)	285 (91.4)	247 (87.0)	0.0849
T2D Onset Can be Delayed with Regular Exercise	479 (80.4)	256 (82.1)	223 (78.5)	0.2785
T2D Onset Can be Delayed with Medication	234 (39.3)	128 (41.0)	106 (37.3)	0.3553
T2D Increases Risk of other Diseases	375 (64.0)	209 (67.9)	166 (59.7)	0.0403
Genetic Literacy: High	514 (86.8)	276 (88.8)	238 (84.7)	0.3429
Genetic Literacy: Average	52 (8.8)	23 (7.4)	29 (10.3)	
Genetic Literacy: Low	26 (4.4)	12 (3.9)	14 (5.0)	
**Worry**				
Worry of acquiring T2D in the future: High	241 (41.2)	148 (48.5)	93 (33.2)	0.0002*
Worry about T2D Genetic Testing: High	154 (26.0)	84 (27.1)	70 (24.7)	0.5124
Worry about Cost of T2D Genetic Testing: High	429 (73.0)	240 (77.4)	189 (67.3)	0.0057
Worry from High Risk Testing Result: High	445 (75.3)	247 (79.7)	198 (70.5)	0.0095
**Motivation**				
Motivation to have T2D Genetic Testing: High	205 (34.4)	185 (59.3)	20 (7.1)	<0.0001*
Motivation to have T2D Genetic Testing: Average	237 (39.8)	106 (34.0)	131 (46.3)	
Motivation to have T2D Genetic Testing: Low	153 (25.7)	21 (6.7)	132 (46.6)	
Motivation Reasons: Want to Know Risk Before T2D Onset	242 (40.6)	186 (59.6)	56 (19.7)	<0.0001*
Motivation Reasons: T2D is Preventable	216 (36.2)	151 (48.4)	65 (22.9)	<0.0001*
Motivation Reasons: Family History	198 (33.2)	141 (45.2)	57 (20.1)	<0.0001*
Motivation Reasons: Know Someone with T2D Complications	156 (26.2)	105 (33.7)	51 (18.0)	<0.0001*
Motivation Reasons: Future Genetic Treatment for T2D	141 (23.7)	105 (33.7)	36 (12.7)	<0.0001*
Motivation Reasons: Personal Risk	71 (11.9)	56 (18.0)	15 (5.3)	<0.0001*
Motivation Reasons: History of Gestational Diabetes	17 (2.8)	13 (4.2)	4 (1.4)	0.0433
Motivation Increase: Insurance Covers Cost	370 (62.1)	221 (70.8)	149 (52.5)	<0.0001*
Motivation Increase: Test is Accurate	312 (52.4)	198 (63.5)	114 (40.1)	<0.0001*
Motivation Increase: Test is Inexpensive	254 (42.6)	150 (48.1)	104 (36.6)	0.0047
Motivation Increase: Physician Recommends Test	213 (35.7)	120 (38.5)	93 (32.8)	0.1459
Motivation Increase: Genetic Test Counseling Provided	205 (34.4)	124 (39.7)	81 (28.5)	0.0040
Motivation Increase: Relative with High Risk Test Result	153 (25.6)	91 (29.2)	62 (21.8)	0.0406
Action Motivation if High Risk Result: Healthier Eating (High/Very)	497 (84.4)	276 (89.0)	221 (79.2)	0.0010
Action Motivation if High Risk Result: Regular Exercise (High/Very)	485 (81.9)	272 (87.5)	213 (75.8)	0.0002*
Action Motivation if High Risk Result: Lose Weight (High/Very)	461 (78.1)	256 (82.5)	205 (73.2)	0.0060
**Utility**				
Discuss T2D Genetic Test (GT) Results with Physician	265 (44.5)	171 (54.8)	94 (33.1)	< .0001*
Uncertainty Clear	234 (39.3)	151 (48.4)	83 (29.2)	< .0001*
Convince Family to get Tested	193 (32.4)	123 (39.4)	70 (24.7)	0.0001*
Test Children as well	157 (26.2)	103 (33.0)	54 (19.0)	0.0001*
Prepare for Future	364 (61.1)	230 (73.7)	134 (47.2)	< .0001*
Start Preventive Actions	351 (59.1)	219 (70.7)	132 (46.5)	< .0001*
**Attitudinal Barriers to Testing**				
Perceived Low Risk	80 (13.4)	18 (5.8)	62 (21.8)	< .0001*
Negative Family History of T2D	64 (10.7)	16 (5.1)	48 (16.9)	< .0001*
Preference for Cancer GT than T2D Genetic Testing	55 (9.2)	15 (4.8)	40 (14.1)	< .0001*
Anticipated Worry from Test Result	47 (7.9)	11 (3.5)	36 (12.7)	< .0001*
Belief: Cannot Change Genetic Risk	42 (7.1)	14 (4.5)	28 (9.9)	0.0105
Lack of Time for T2D Genetic Testing	28 (4.7)	8 (2.6)	20 (7.0)	0.0099
Poor Access to Health Care	25 (4.2)	6 (1.9)	19 (6.7)	0.0037
Distrust in T2D Genetic Testing Results	17 (2.9)	1 (0.32)	16 (5.6)	< .0001*
Harmful	14 (2.4)	5 (1.6)	9 (3.2)	0.2073
**Trust**				
Availability of T2D Genetic Testing: Yes	472 (80.8)	273 (88.9)	199 (71.8)	< .0001*
Consideration of High Risk Test Result: High T2D Risk	487 (83.3)	262 (85.3)	225 (80.9)	0.1541
**Intention**				
Change in Willingness: Testing is Cost-free	442 (74.3)	302 (96.8)	140 (49.5)	< .0001*
Change in Willingness: Knowing T2D is Preventable	482 (81.0)	304 (97.4)	178 (62.9)	< .0001*

*P-value remains significant after Bonferonni correction; p<0.007.

When asked to rate reasons to consider T2D genetic testing, individuals rated the desire to know risk prior to diagnosis as the most common reason (40.6%; Table 2) followed by: knowledge that T2D is a preventable disease (36.2%), positive family history for T2D (33.2%), knowing someone with T2D related complications (26.2%), possibility of gene therapy for T2D in the future (23.7%), and high perceived personal risk (11.9%). Each of these reasons significantly distinguished those with and without interest in genetic testing (p’s < .0001). Reasons that increased motivation to engage in genetic testing were insurance coverage for the costs of testing, and knowledge that the genetic test is accurate (p’s < .0001). Individuals interested in genetic testing reported high/very high motivation to exercise (p = 0.0002).

Participants considered the test to be useful if the test results would lead them to do any of the following: discuss their test results with their physician(s), change their level of risk, convince their family member(s) to get tested, become motivated to have their children tested, better prepare themselves for the future, and initiate preventive behaviors (p’s<0.0001; Table 2). The most common reason to reject genetic testing was low perceived risk for developing T2D in the future (10.4%, p<0.0001). Other psychosocial barriers (Table 2) to testing were: absence of family history of T2D, prefer genetic testing for cancer risk prediction, anticipated worry about the test results, and distrust of the test results (p’s<0.0001).

Participants were asked if their interest in genetic testing would change based on the test being free of cost or T2D is preventable (p’s < .0001, Table 2). Among individuals not interested in testing (n = 285), 50% became interested if the test was free of cost and 64% became interested if they were told that T2D is preventable.

### Factors Influencing Decisions to Engage in T2D Genetic Testing: Multivariable Analyses

Variables associated with interest in pursuing T2D genetic testing in the univariate models were entered into multiple logistic regression analyses and were adjusted for age, gender, BMI and recruitment location (Table 3). Psychosocial predictors that remained significantly associated with increased interest in genetic testing were motivation, behavioral intention and trust. Specifically, motivation associated with high perceived personal risk of T2D [OR = 4.38 (1.76, 10.9)], motivation associated with family history of T2D [OR = 2.56 (1.46, 4.48)], motivation to engage in testing associated with a desire to know risk prior to disease onset [OR = 3.25 (1.94, 5.42)], motivation associated with knowledge that T2D is a preventable disease [OR = 2.11 (1.24, 3.60)], and increased intention for genetic testing if free of cost [OR = 10.2 (4.27, 24.6)] or T2D is preventable [5.18 (1.95, 13.7)]. Distrust of the results of genetic testing significantly decreased interest in testing [OR = 0.03 (0.003, 0.30)].

**Table 3:** Psychosocial Predictors of Willingness to Consider Type 2 Diabetes Genetic Testing: Multivariable Analysis.

Psychosocial Predictor	OR (95% CI)^a^	P-value
Reason for motivation: Individual is high risk (Yes)	4.38 (1.76, 10.9)	0.0015
Reason for motivation: Family history (Yes)	2.56 (1.46, 4.48)	0.0010
Reason for motivation: Prefer to know risk before diagnosis (Yes)	3.25 (1.94, 5.42)	< .0001
Reason for motivation: Diabetes is preventable (Yes)	2.11 (1.24, 3.60)	0.0061
Disagree: Do not trust genetic testing results (Yes)	0.03 (0.003, 0.30)	0.0029
Intention: Genetic testing is free of cost (Yes)	10.2 (4.27, 24.6)	< .0001
Intention: T2D is preventable (Yes)	5.18 (1.95, 13.7)	0.0010

^a^Adjusting for Age, BMI, Gender and Recruitment location

## Discussion

This study examined demographic and psychosocial factors that predict individual interest in genetic testing for T2D. Findings from this study build on an emerging literature that has begun to characterize attitudes about genetic testing for chronic diseases and T2D specifically in the context of an epidemic of T2D among adults and children. Our findings indicate overall receptivity and interest in genetic testing for T2D, particularly among those who feel the test is most relevant to their health status (i.e. high perceived personal risk).

Participants demonstrated relatively high levels of knowledge about genetic testing (genetic literacy) and awareness that the risk of T2D is increased if there is a family history of the disease. We observed that preference to know risk prior to diagnosis of T2D and the ability to prevent T2D were significant predictors of interest in testing. In addition, public distrust of the test emerged as the sole factor predicting rejection of genetic testing for T2D. This suggests that similar to other forms of health screening, the onus remains on medical and behavioral science to demonstrate the value of this tool to a generally receptive public. In addition, our data demonstrated that when individuals were told that the cost of testing was free and that T2D can be prevented, interest in genetic testing increased.

We further observed that only 57% of the sample demonstrated knowledge that T2D is preventable. More than a decade since the release of the first Diabetes Prevention Program outcomes paper[24], there remains a significant gap in knowledge between health care professionals and the public about the ability to prevent this chronic disease. Beyond the public health implications about diabetes prevention, this gap also has implications for the adoption of genetic testing. Data from this study suggests that genetic testing for T2D is more highly valued when individuals know there is a way to prevent T2D.

Risk factors such as family history, BMI and metabolic status are currently used by providers in routine care to advise patients about their risk of developing T2D. Our data suggests genetic testing for T2D may be most utilized once individuals have been identified as having high risk using conventional risk factors. These data also point to the public’s awareness of the accuracy of medical tests, which will have to be reported clearly when individuals are considering genetic testing. Likewise the data suggest that the public is more amenable to testing if any cost incurred by them is eliminated. Finally, these findings demonstrate a disparity between public knowledge about T2D prevention and interventions that have been empirically demonstrated to achieve these goals.

These data, along with previous studies, demonstrates public receptivity to genetic testing among those with and at risk for T2D. One implication for primary care medicine is that providers can feel confident that their patients are likely to be receptive to genetic testing for risk assessment. At the same time, primary care providers should be aware that their patients may be utilizing commercial venues that measure their genetic risk only and without their clinical risk has limited predictive ability. When rigorous and reliable genetic testing information becomes available, there will be an opportunity for primary care providers to use this information as a teachable moment and to connect patients to diabetes prevention and education in their community.

Studies investigating characteristics associated with interest in genetic testing to predict risk of developing T2D or chronic diseases that include T2D are limited. The strengths of our study are the comprehensive measures of the many psychological constructs related to behavior change[9–11, 16, 25, 26]; and purposely recruiting individuals representing greater ethnic and socioeconomic diversity [9, 16, 25, 26].

Limitations of this study included the use of a convenience sample and self-reported risk factors. Although use of a convenience sample may limit the generalizability of findings, our large sample size (n = 598) contained substantial variance across demographic characteristics including age, gender, race, ethnicity, and socio-economic status suggesting that we captured a broad cross-section of adults. In addition, all data was self-reported, including T2D status. BMI was estimated from self-reported height and weight and should be interpreted as approximations to actual values.

Advances in genetic discovery coupled with the decreasing costs of sequencing an individual’s entire genome have set the stage for personalized medicine to improve health outcomes. There is a small and growing group of physicians who constitute ‘early adopters’ of this source of information who have begun integrating patient’s genetic information into their clinical care. Further work is needed to make genetic testing for T2D useful for widespread use by primary care providers. An expanding number of alleles associated with the risk for T2D requires more discovery research to determine the individual and combined contribution of these SNPs on disease subtypes, T2D initiation and outcomes. In advance of refined prediction, it is possible that T2D genetic risk testing may have the most utility in high-risk patient groups. T2D genetic risk testing may also evolve for use as an intervention early in life to prevent the onset of the risk factors (i.e. weight, dietary and physical activity behaviors) or to tailor prevention and treatment options to the individual. Research is needed to develop and test the efficacy and effectiveness of this work with the goal of reducing the overall burden of T2D in the general population.

## Supporting Information

S1 Appendix Questionnaire. (DOCX)
