# Supplementary material for: Factors Motivating Individuals to Consider Genetic Testing for Type 2 Diabetes Risk Prediction
Source: PLoS One. 2016 Jan 20;11(1):e0147071. doi: 10.1371/journal.pone.0147071 (PMC4720283; doi:10.1371/journal.pone.0147071)
Supplement: S1 Appendix — (DOCX) [file pone.0147071.s001.docx]

# S1 Appendix. Questionnaire.

1) What is your age (in years)?

____________________________________________

2) Your height in feet-inches is: (e.g. 5'-4")

____________________________________________

3) Your weight (in pounds) is:

____________________________________________

4) What is your gender?

- Female
- Male

5) What is your current marital status?

- - Married
  - Widowed
  - Divorced
  - Separated
  - Never married, single
  - Living with a partner, not married

6) Do you have at least one child who is below 18 years of age?

- Yes
- No

7) Which option represents your annual household income level?

- Less than $25,000
- $25,000 - $50,000
- $50,001 - $75,000
- $75,001 - $100,000
- More than $100,000

8) Which option best describes your current health insurance?

- Medicaid or CHIP
- Medicare
- Commercial private insurance
- Do not have health insurance

9) Your highest education level is:

- Less than High School
- Some High School, but did not finish
- Completed High School
- College, but no degree
- College with Associate's or Bachelor's degree
- Master's or Professional or Doctorate degree, enrolled or completed

10) Are you Latino or Hispanic?

- Yes
- No

11) Which of the following best describes your race?

- American Indian or Alaska Native
- Black or African-American
- Asian
- Native Hawaiian or other Pacific Islander
- White or Caucasian
- Prefer not to answer
- Other

12) If checked 'other' in the above question, please specify:

____________________________________________

**Answer the next question if you are a woman:**

13) Are you currently pregnant?

- Yes
- Not sure
- No

**Now we would like to ask you about your health and your family's health:**

14) In general, you would describe your health as:

- Excellent
- Very Good
- Good
- Fair
- Poor

15) Which of the following conditions do you have or have had in the past? (Check all that apply)

- Type 1 diabetes or juvenile diabetes
- Type 2 or adult-onset diabetes
- High blood pressure or hypertension
- High blood sugar, but not diabetes
- High blood cholesterol or other lipids
- Coronary heart disease
- Overweight
- Obesity
- Pregnancy-related diabetes or gestational diabetes
- None of the above

16) Does type 2 diabetes run in your family (blood relatives)?

- Yes
- No
- Don't know or not sure

**The following questions are related to your knowledge of type 2 diabetes:**

17) Which options do you feel are true for type 2 diabetes? (Check all that apply)

- Type 2 diabetes can be cured
- Type 2 diabetes can be prevented
- Type 2 diabetes is entirely inherited
- Type 2 diabetes is partly inherited and partly due to lifestyle conditions
- The risk of getting type 2 diabetes cannot be changed
- The risk of getting type 2 diabetes can be changed
- The onset of type 2 diabetes can be delayed

18) If you get type 2 diabetes, then would you have an increased risk for other diseases too?

- Yes
- Not sure
- No

19) Which option best explains how your life would change if you get type 2 diabetes?

- Drastic change, and difficult to manage day-to-day living
- Considerable change, however, manageable day-to-day living
- Slight change, and manageable day-to-day living
- No change from the present, and you currently do not have type 2 diabetes
- No change from the present, and you currently have type 2 diabetes

20) Which options do you think can delay the onset of type 2 diabetes? (Check all that apply)

- Healthier eating
- Regular exercise
- Medication
- Cannot delay the onset of type 2 diabetes
- Other, please specify: ______________

21) On a scale of 1 to 7, how likely do you think you will get type 2 diabetes in the future?

- 1 Extremely Unlikely
- 2
- 3
- 4 Neither likely nor unlikely
- 5
- 6
- 7 Extremely Likely

22) On a scale of 1 to 7, how worried are you that you will get type 2 diabetes in the future?

- 1 Not Worried at all
- 2
- 3
- 4 Somewhat Worried
- 5
- 6
- 7 Extremely Worried

**Now we would like to learn about your experience and knowledge of genetic testing:**

23) Had you heard or read about genetic testing before receiving this questionnaire?

- Yes
- No

24) Your knowledge of genetic diseases and genetic testing for diseases is mainly from: (Check all that apply)

- Media (e.g. internet, television, magazines, posters)
- Physician
- Family
- Friends
- Teachers
- Other, please specify: ___________

25) Have you ever had genetic testing done in the past?

- Yes
- No
- Don't know or not sure

**Answer the next 2 questions if you answered 'yes' in the above question:**

26) For what disease or diseases did you get genetic testing done?

____________________________________________

____________________________________________

27) Which option best describes your past experience with genetic testing?

- Very good
- Good
- Average
- Bad
- Very bad

**Now we would like to know your opinion about genetic testing specific to type 2 diabetes:**

28) To what extent do you agree that a genetic test should be available to people that can predict their risk for getting type 2 diabetes?

- Strongly Agree
- Agree
- Neither Agree nor Disagree
- Disagree
- Strongly Disagree

29) If a genetic test shows that you are at a high risk for getting type 2 diabetes, you believe your actual risk for type 2 diabetes would be:

- High
- Medium
- Low
- None

**The questions below will help us understand your willingness to have genetic testing for diabetes risk:**

30) Would you like to get genetic testing done for type 2 diabetes?

- Yes
- Not sure
- No

31) Your motivation to have genetic testing for type 2 diabetes is:

- Very High
- High
- Average
- Low
- Very Low

32) You are currently motivated to have genetic testing for type 2 diabetes because: (check all that apply)

- You have a high risk for type 2 diabetes
- Type 2 diabetes runs in your family
- You prefer to know your diabetes risk before you are diagnosed with the disease
- You know someone who has complications of diabetes
- You had diabetes during pregnancy
- You believe type 2 diabetes can be prevented
- In the future, there may be a genetic treatment for type 2 diabetes
- Other, please specify: ____________________________
- You are not motivated towards genetic testing for type 2 diabetes

33) Knowing your type 2 diabetes genetic test results will be useful to you because: (check all that apply)

- Uncertainty of your diabetes risk will become clearer
- You can discuss your genetic test results with your physician
- You can convince your family to be tested too
- You can better prepare yourself for the future
- Your test results will motivate you to take actions to decrease your diabetes risk
- You would know if your children should get tested too
- Other, please specify: __________________________
- Knowing your genetic test results for type 2 diabetes will not be useful to you

34) You currently disagree with getting genetic testing for type 2 diabetes because: (check all that apply)

- You are already at a low risk for type 2 diabetes
- Type 2 diabetes does not run in your family
- You cannot change your genetic risk for type 2 diabetes
- You would rather get tested for cancer than for type 2 diabetes
- Genetic testing for type 2 diabetes can be harmful for you
- You would feel more worried if your genetic test result showed a high risk for type 2 diabetes
- You do not have access to good health-related facilities and services
- You would not trust the genetic test results for type 2 diabetes
- You do not have time to get genetic testing done
- Other, please specify: __________________
- You currently agree to get genetic testing done for type 2 diabetes

35) Which options would further increase your motivation towards genetic testing for type 2 diabetes? (check all that apply)

- Your insurance covers the cost of testing
- The genetic test is not expensive for you
- Pre-test and post-test counseling is available for you
- The test is known to be accurate in predicting your diabetes risk
- Your physician recommends you to take the test
- Your family member has a high risk for type 2 diabetes, based on genetic test results
- Other, please specify: _____________________
- You are not motivated towards genetic testing for type 2 diabetes

36) You would agree to get a type 2 diabetes genetic testing done if it is recommended by: (check all that apply)

- Physician
- Genetic counselor
- Media (e.g. internet, television, magazines, posters) Family member
- Friends
- Other, please specify: ________

37) How would your religious faith affect your willingness towards genetic testing for type 2 diabetes?

- Strongly increase your willingness
- Increase your willingness
- Neither increase nor decrease your willingness
- Decrease your willingness
- Strongly decrease your willingness

38) How would your cultural background affect your willingness towards genetic testing for type 2 diabetes?

- Strongly increase your willingness
- Increase your willingness
- Neither increase nor decrease your willingness
- Decrease your willingness
- Strongly decrease your willingness

39) Assuming that genetic testing is free of cost, would you now want genetic testing done for type 2 diabetes?

- Yes
- No
- Not sure

40) If your answer to the above question is 'no' or 'not sure', then your reasons are:

41) Assuming that type 2 diabetes is completely preventable, would you now want genetic testing done for type 2 diabetes?

- Yes
- No
- Not sure

42) If your answer to the above question is 'no' or 'not sure', then your reasons are:

**The questions below will help us understand how worried you would be on a scale of 1 to 7 about genetic testing for type 2 diabetes:**

43) On a scale of 1 to 7, how worried are you to have your genetic testing done for type 2 diabetes?

- 1 Not Worried at all
- 2
- 3
- 4 Somewhat Worried
- 5
- 6
- 7 Extremely Worried

44) How worried would you be about the cost of genetic testing?

- 1 Not Worried at all
- 2
- 3
- 4 Somewhat Worried
- 5
- 6
- 7 Extremely Worried

45) How worried would you be if the genetic test analyzes a sample of your saliva (spit)?

- 1 Not Worried at all
- 2
- 3
- 4 Somewhat Worried
- 5
- 6
- 7 Extremely Worried

46) How worried would you be if the genetic test analyzes your blood sample?

- 1 Not Worried at all
- 2
- 3
- 4 Somewhat Worried
- 5
- 6
- 7 Extremely Worried

47) How worried would you be if your genetic test results predict that you have a high risk of getting type 2 diabetes in the future?

- 1 Not Worried at all
- 2
- 3
- 4 Somewhat Worried
- 5
- 6
- 7 Extremely Worried

48) How worried would you be if your genetic test results predict that you have a low risk of getting type 2 diabetes in the future?

- 1 Not Worried at all
- 2
- 3
- 4 Somewhat Worried
- 5
- 6
- 7 Extremely Worried

**Now we would like to know how the genetic test results for type 2 diabetes would affect your lifestyle:**

49) If your genetic test result indicates a high risk for type 2 diabetes, your motivation to adopt healthier eating habits would be:

- Very High
- High
- Average
- Low
- Very Low

50) If your genetic test result indicates a high risk for type 2 diabetes, your motivation to exercise regularly would be:

- Very High
- High
- Average
- Low
- Very Low

51) If your genetic test result indicates a high risk for type 2 diabetes, your motivation to lose weight would be:

- Very High
- High
- Average
- Low
- Very Low

**The following true and false questions are related to your knowledge of 'genetics':**

52) Genes are parts of your DNA that can predict your risk for specific diseases

- True
- False

53) Both the genes and the environment contribute to risk for diseases

- True
- False

54) It is possible for healthy biological parents to have a child with a hereditary disease

- True
- False

55) Some heritable diseases express themselves later in adult life

- True
- False

56) If a person is genetically predisposed for a disease, this person will certainly get the disease

- True
- False
